# Identifying multi-compartment Hodgkin-Huxley models with high-density extracellular voltage recordings

**Published:** 2025-06-25

**Authors:** Ian Christopher Tanoh, Michael Deistler, Jakob H. Macke, Scott W. Linderman

## Abstract

Multi-compartment Hodgkin-Huxley models are biophysical models of how electrical signals propagate throughout a neuron, and they form the basis of our knowledge of neural computation at the cellular level. However, these models have many free parameters that must be estimated for each cell, and existing fitting methods rely on intracellular voltage measurements that are highly challenging to obtain in vivo. Recent advances in neural recording technology with high-density probes and arrays enable dense sampling of extracellular voltage from many sites surrounding a neuron, allowing indirect measurement of many compartments of a cell simultaneously. Here, we propose a method for inferring the underlying membrane voltage, biophysical parameters, and the neuron's position relative to the probe, using extracellular measurements alone. We use an Extended Kalman Filter to infer membrane voltage and channel states using efficient, differentiable simulators. Then, we learn the model parameters by maximizing the marginal likelihood using gradient-based methods. We demonstrate the performance of this approach using simulated data and real neuron morphologies.

### 1 Introduction

Biological neurons are equipped with a wide range of biophysical mechanisms that contribute to information processing [Poirazi et al., 2003a, Spruston, 2008, Gidon et al., 2020] and to the brain's robustness to environmental perturbations [O'Leary et al., 2013, Haddad and Marder, 2018]. Multi-compartment Hodgkin-Huxley models provide a principled framework for studying biophysical mechanisms of neurons [Hodgkin and Huxley, 1952, Carnevale and Hines, 2006]. Multi-compartment models treat neurons as connected electrical compartments, allowing simulation of how voltage signals propagate throughout a cell as ion channels open and close. A central limitation for building multi-compartment models—and for understanding biophysical mechanisms in neurons—is the lack of experimental techniques to precisely measure the biophysical parameters of these models. As such, these parameters must be inferred from recordings of the activity of neurons [Huys et al., 2006, Mensi et al., 2012, Almog and Korngreen, 2014,

[1] Department of Statistics and the Wu Tsai Neurosciences Institute, Stanford University, Stanford, CA, USA
[2] Machine Learning in Science, University of Tübingen, Germany
[3] Tübingen AI Center, Tübingen, Germany
[4] Department of Empirical Inference, Max Planck Institute for Intelligent Systems, Tübingen, Germany
Correspondence: `ictanoh@stanford.edu` and `scott.linderman@stanford.edu`

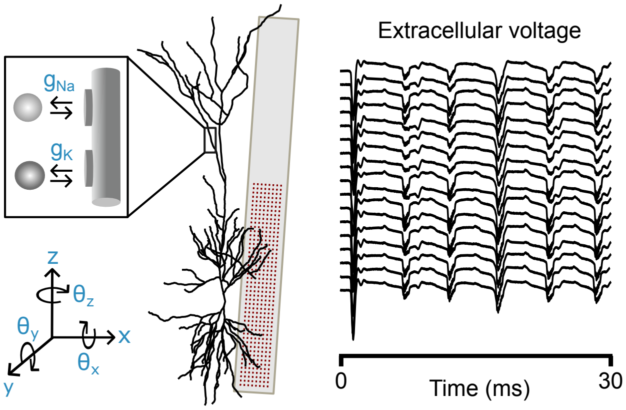

*Figure 1:* **Our goal: Inferring biophysical properties of single neurons from dense extracellular measurements:** We want to identify key biophysical properties—maximum membrane conductances of ion channels and the cell's relative position to the probe (left, blue) from extracellular voltage traces recorded from multiple sites along the probe (right), capturing the neuron's activity over time.

Gonçalves et al., 2020]. Typically, these recordings are made from within a neuron using intracellular measurements such as patch-clamp electrophysiology, which enables precise recordings of voltage dynamics in the soma [Sakmann and Neher, 1984, Hamill et al., 1981] and, for large neurons, in dendrites [Stuart and Sakmann, 1994]. However, intracellular methods are technically challenging, and only provide voltage signals from a very small number of locations within a neuron (typically, only from the soma) [Margrie et al., 2002].

Recent advances in neural recording technologies have opened new possibilities for studying neuron biophysics *in vivo*. High-density silicon probes such as the Neuropixel Ultra [Ye et al., 2024] can record extracellular voltage traces across hundreds of closely spaced electrodes. These signals, which reflect the transmembrane currents generated by nearby neurons, are traditionally used for spike detection and sorting—identifying when and where neurons fire spikes [Pachitariu et al., 2016, Rossant et al., 2016].

While effective for tracking population activity, this approach treats spikes as discrete events and ignores the exquisite biophysical details of neurons [Einevoll et al., 2012].

In this work, we revisit the extracellular signal, not just as a source of discrete spike times, but as a rich, spatially structured observation from which we can infer biophysical properties of individual neurons. This is made possible by three key developments: (1) high-density probes and 2D arrays that allow us to sample extracellular voltage at many sites around a cell to triangulate its position and morphology; (2) differentiable simulators like JAXLEY [Deistler et al., 2024] that enable fast, hardware-accelerated simulation of complex biophysical models and gradient-based parameter optimization; and (3) scalable state-space inference frameworks such as DYNAMAX [Linderman et al., 2025], which allow efficient state inference and parameter estimation in high-dimensional, nonlinear dynamical systems.

We leverage these developments to provide a statistical approach for inferring the parameters of multi-compartment biophysical models from extracellular observations: We extend classical multi-compartment Hodgkin–Huxley models to include a biophysical model of extracellular potentials. We then use an Extended Kalman Filter (EKF) [Särkkä and Svensson, 2023] to infer latent neuronal states—such as membrane voltages and gating variables—from observed extracellular signals and we improve the computational efficiency with a diagonal approximation to the filtering covariance. To estimate model parameters, including ion channel densities and the neuron's spatial position relative to the probe, we maximize the marginal likelihood using gradient-based optimization.

We first demonstrate that our method can successfully infer membrane properties and cell locations in simplified neuron models, and that the use of an EKF largely improves the method's robustness to model misspecification. We then apply our method to synthetic extracellular data generated from real neuron morphologies and demonstrate that the method can scale to morphologies with more than a hundred compartments. Overall, our results suggest that the combination of differentiable simulation and state-space modeling makes it possible to estimate biophysical properties across the entire morphology of a neuron based only on extracellular voltage recordings.

## 2 Biophysical Modeling of Neurons

### 2.1 Modeling Action Potentials with Hodgkin–Huxley Dynamics

The multi-compartment Hodgkin–Huxley model (HH) describes how ion channels shape the membrane potential across branches of a neuron. Each compartment represents a small component of the cell with its own voltage, capacitance, and set of ion channels. Let $V^{(i)}$ denote the membrane potential of compartment $i$, with surface area $S^{(i)}$ and specific membrane capacitance $C_m$. Each compartment has a set of ion channels, $\mathscr{C}$, and each channel $c \in \mathscr{C}$ is characterized by a maximum conductance $g_c^{(i)}$, reversal potential $E_c$, and a time-varying state $\lambda_c^{(i)}$. A nonlinear activation function, $a_c(\lambda_c^{(i)}) \in [0,1]$, specifies the fraction of the maximum current that can flow through the channel, as a function of its state. The voltage dynamics for compartment $i$ are given by,

$$C_m \frac{\mathrm{d}V^{(i)}}{\mathrm{d}t} = \frac{I_{\text{ext}}^{(i)}}{S^{(i)}} - \sum_{c \in \mathscr{C}} g_c^{(i)} a_c(\lambda_c^{(i)})(V^{(i)} - E_c) + \sum_{j \sim i} g^{(i,j)}(V^{(j)} - V^{(i)}), \tag{1}$$

where $I_{\text{ext}}^{(i)}$ is the external current applied to compartment $i$, and $g^{(i,j)}$ is the axial conductance coupling compartment $i$ to its neighbor $j$. The total transmembrane current in compartment $i$ is denoted by $I_m^{(i)} = \sum_{c \in \mathscr{C}} g_c^{(i)} a_c(\lambda_c^{(i)})(V^{(i)} - E_c)$. For instance, in the original single compartment Hodgkin–Huxley model with sodium, potassium, and leak channels, the total current is,

$$I_m = g_{\text{Na}}\, m^3 h(V - E_{\text{Na}}) + g_{\text{K}}\, n^4(V - E_{\text{K}}) + g_{\text{L}}(V - E_{\text{L}}). \tag{2}$$

Here, for example, the states of the sodium channel are $\lambda_{\text{Na}} = (m,h) \in [0,1]^2$ and the activation function is $a_{\text{Na}}(\lambda_{\text{Na}}) = m^3 h$. The gating variables evolve according to first-order kinetics $\frac{\mathrm{d}\lambda_c}{\mathrm{d}t} = \alpha_c(V)(1 - \lambda_c) - \beta_c(V)\lambda_c$, where $\alpha_c(V)$ and $\beta_c(V)$ are voltage-dependent rate functions that control the opening and closing transitions of the ion channel. Specific forms for the $\alpha$ and $\beta$ functions are available in Dayan and Abbott [2005], e.g.

### 2.2 Modeling Extracellular Voltage Measurements

Extracellular voltages reflect the collective activity of nearby neurons. While the physics of extracellular recordings are well understood [Gold et al., 2006, Lindén et al., 2014], our work is, to our knowledge, the first to leverage this framework for fitting multi-compartment Hodgkin–Huxley models to extracellular measurements. Let $\Phi$ denote the extracellular potential measured by a fixed site on a recording probe. We treat each compartment as a point source of transmembrane current located at its center and denote $r_i$ the distance from the recording site to compartment $i$ of a neuron. It follows from Coulomb's law that the measured extracellular voltage is,

$$\Phi \propto \sum_{i=1}^{N} \frac{I_m^{(i)}}{r_i}. \tag{3}$$

In words, the observed potential $\Phi$ is proportional to the sum of transmembrane currents from nearby compartments, weighted by the inverse of the distance to the compartment.

## 3 State Inference and Parameter Estimation

We aim to infer the membrane potential and channel states and to estimate the biophysical and structural parameters of a neuron from noisy extracellular voltage traces. We can achieve both goals by casting the multi-compartment Hodgkin-Huxley model as a state space model (SSM) with nonlinear dynamics. However, the nature and scale of these models present unique challenges, which we address below.

### 3.1 State Space Model Formulation

A state space model (SSM) is a latent variable model with latent states $\mathbf{z}_{1:T}$, observations $\mathbf{y}_{1:T}$, and parameters $\theta$. In a Markovian SSM, latent states evolve according to a dynamics distribution, $p(\mathbf{z}_t \mid \mathbf{z}_{t-1}; \theta)$, and they give rise to observations via an emission distribution $p(\mathbf{y}_t \mid \mathbf{z}_t; \theta)$. There are a host of well-established techniques for state inference and parameter estimation in such models [Särkkä and Svensson, 2023, Linderman et al., 2025], depending on the nature of the dynamics and emission distributions.

In our case, the model parameters, $\theta$, may include the maximum conductances of various ion channels as well as parameters governing the neuron's spatial configuration relative to the recording probe. The latent states consist of the voltages and channel states for each compartment, $\mathbf{z}_t = [V^{(1)}, \dots, V^{(N)}, \{\lambda_c^{(1)}\}_{c \in \mathscr{C}_1}, \dots, \{\lambda_c^{(N)}\}_{c \in \mathscr{C}_N}]$. To model the state dynamics, we use an Euler discretization of the multi-compartment Hodgkin-Huxley differential equations (see eq. (1)),

$$\mathbf{z}_{t+1} \sim \mathscr{N}(f_\theta(\mathbf{z}_t, t),\, dt\, \Sigma_{\text{dyn}}), \tag{4}$$

where $f_\theta(\mathbf{z}_t, t)$ is the deterministic update function derived from the ODEs above, and $\Sigma_{\text{dyn}}$ is a diagonal covariance matrix with entries from $\{\sigma_v^2,\ \sigma_{\text{state}}^2\}$. This Gaussian noise is intended to account for model uncertainty, such as missing ion channels or misspecified channel dynamics, which we will consider in the results section. Since the gating variables are constrained to the interval $[0, 1]$, they are transformed using the logit function. For simplicity, we also refer to the transformed vector as $\mathbf{z}_t$.

We assume that the only source of stochasticity in the emission process is measurement noise. Let $\mathbf{y}_t \in \mathbb{R}^M$ denote the observed extracellular potential at time $t$ across $M$ recording sites. It is modeled as,

$$\mathbf{y}_t \sim \mathscr{N}(h_\theta(\mathbf{z}_t), \sigma_{\text{obs}}^2 I), \tag{5}$$

where $h_\theta(\mathbf{z}_t)$ is the predicted extracellular voltage from eq. (3) and $\sigma_{\text{obs}}^2$ is the observation noise variance. Since $h_\theta(\mathbf{z}_t)$ only depends on $\mathbf{z}_t$, the emission model is conditionally independent of previous states given the current latent state.

#### 3.2 Scaling the Extended Kalman Filter to Large Biophysical Models

Since the dynamics and emission models are nonlinear, there is no closed-form solution for the filtering distribution $p_\theta(\mathbf{z}_{1:t} \mid \mathbf{y}_{1:t})$. To address this challenge, we use the extended Kalman filter (EKF), which linearizes the dynamics and emission functions via first-order Taylor expansions [Särkkä and Svensson, 2023]. This linear approximation enables recursive estimation of the latent states, and it also provides an estimate of the marginal likelihood $p_\theta(\mathbf{y}_{1:T})$ for parameter estimation.

Biologically realistic neuron models often contain dozens or even hundreds of compartments, leading to latent state dimensions in the hundreds or thousands. The standard EKF maintains an estimate of the full state covariance matrix, and updating the filtering distribution requires solving a linear system with this matrix. Since this computation scales cubically with the number of states, it becomes computationally infeasible to apply standard EKF formulations to full-scale neuronal models. To overcome this challenge, we develop a scalable EKF variant for biophysical neuron model fitting. Our approach leverages a diagonal approximation of the posterior covariance, inspired by Chang et al. [2022], as well as the special structure of multi-compartment Hodgkin-Huxley dynamics.

We approximate the EKF filtering distribution $p_t(x) = \mathcal{N}(x; \mu_t, \mathbf{\Sigma}_t)$ with a *diagonal* Gaussian distribution $q_t(x) = \mathcal{N}(x; \tilde{\mu}_t, \tilde{\mathbf{\Sigma}}_t)$ that minimizes the KL divergence $\mathrm{KL}(q_t \,\|\, p_t)$. This divergence minimization yields a closed-form solution: we match the marginal means and precisions (inverse covariances) of $p_t$ and $q_t$. The resulting diagonal covariance estimate is given by

$$\tilde{\mathbf{\Sigma}}_t = \mathrm{diag}\left(\left[\left(\mathbf{\Sigma}^{ii}_{t|t-1}\right)^{-1} + \left(\mathbf{H}_t^T \mathbf{R}_t^{-1} \mathbf{H}_t\right)_{ii}\right]^{-1}\right), \tag{6}$$

where $\mathbf{\Sigma}_{t|t-1}$ is the predicted covariance from the EKF prediction step, $\mathbf{R}_t = \sigma^2_{\text{obs}} I$ is the observation noise covariance, and $\mathbf{H}_t = \mathrm{Jac}(h_\theta)(\mu_{t \mid t-1})$ is the Jacobian of the emission function $h_\theta$ evaluated at the predicted state $\mu_{t \mid t-1}$.

Importantly, the diagonal of $\mathbf{H}_t^T \mathbf{R}_t^{-1} \mathbf{H}_t$ can be computed in linear time with respect to the number of states. The remaining challenge is to efficiently compute the diagonal of the predictive covariance, $\mathbf{\Sigma}_{t|t-1}$, given by

$$\mathbf{\Sigma}_{t|t-1} = \mathbf{F}_t \mathbf{\Sigma}_{t-1} \mathbf{F}_t^T + \mathbf{Q}_t, \tag{7}$$

where $\mathbf{F}_t = \mathrm{Jac}(f_\theta)(\mu_{t \mid t-1})$ is the Jacobian of the dynamics function $f_\theta$ evaluated at the predicted state $\mu_{t \mid t-1}$, and $\mathbf{Q}_t = dt\Sigma_{\text{dyn}}$ is the dynamics covariance matrix.

Chang et al. [2022] assumed $\mathbf{F}_t$ to be the identity matrix, simplifying the diagonal computation. In contrast, our dynamics are nonlinear, and $\mathbf{F}_t$ is nontrivial. However, assuming that $\mathbf{\Sigma}_{t-1}$ is diagonal, we can leverage the sparsity of $\mathbf{F}_t$ to compute the diagonal of $\mathbf{\Sigma}_{t \mid t-1}$ efficiently. In particular, while voltage updates may depend on all other states due to the implicit Euler solver used in JAXLEY [Deistler et al., 2024], gating variables typically depend only on their own previous value and the voltage of their corresponding compartment. This localized structure implies that $\mathbf{F}_t$ is sparse and structured, allowing us to reduce the cost of computing the diagonal of $\mathbf{\Sigma}_{t \mid t-1}$ from quadratic to linear in the number of states (see Appendix F). Importantly, we do not compute the full dense Jacobian $\mathbf{F}_t$; instead, we use the SPARSEJAC library [Schubert, 2023], which leverages graph coloring strategies to efficiently compute sparse Jacobians given a known sparsity pattern. This approach significantly reduces memory usage and computation time, making large-scale EKF updates tractable. With both diagonal components available in linear time, we can compute $\tilde{\boldsymbol{\Sigma}}_t$ efficiently, enabling scalable approximate EKF updates for large models.

Finally, to optimize model parameters, we perform gradient ascent on the marginal likelihood using the Adam optimizer [Kingma and Ba, 2014]. We obtain an estimate for the marginal likelihood based on the EKF approximation,

$$p(\mathbf{y}_{0:T}) \approx \mathcal{N}(\mathbf{y}_0; \mu_0, \Sigma_0) \prod_{t=1}^{T} \mathcal{N}\left(\mathbf{y}_t; h_\theta(\mu_{t|t-1}), \mathbf{H}_t \boldsymbol{\Sigma}_{t|t-1} \mathbf{H}_t^T + \mathbf{R}_t\right), \tag{8}$$

where $\mu_0$ and $\Sigma_0$ denote the initial state mean and covariance matrix respectively. Since we approximate $\boldsymbol{\Sigma}_{t \mid t-1}$ by its diagonal to construct $\tilde{\boldsymbol{\Sigma}}_t$, we also substitute this diagonal approximation into eq. (8) to estimate the marginal likelihood. Performing gradient ascent on the marginal likelihood requires to obtain the gradient through the biophysical model. To obtain this gradient, we use the JAXLEY toolbox, a differentiable simulator of multi-compartment neuron models. All code used for inference, cell and voltage simulation, and the experiments presented in this paper is available at: `https://github.com/ianctanoh/eap-fit-hh`. Details of the computational resources used in this work are provided in Appendix A.

## 4 Validation on simple morphologies

### 4.1 Validation on single-compartment neuron model

We first validated our method on simple synthetic neuron morphologies. We considered a single-compartment neuron equipped with sodium and potassium Hodgkin–Huxley channels. The detailed experimental setup is provided in Appendix B.1. We initially assumed that the biophysical model was correctly specified and that the dynamics were fully deterministic. The extracellular probe had a single recording site placed 10$\mu$m from the neuron, and the cell was stimulated with constant current injection. Our goal was to infer the maximum conductances, assuming the cell's position was known. We simulated 40 extracellular voltage recordings corrupted by Gaussian noise with a standard deviation of 3$\mu$V, reflecting a high signal-to-noise scenario. Maximum conductances were initialized uniformly within biologically plausible ranges. EKF-based inference accurately recovered the true parameters across multiple random seeds, with low variance and negligible bias (fig. 2A), supporting the validity of our approach.

Next, we assessed the effect of observation noise. Neuropixels Ultra probes exhibit RMS noise levels around 7.4$\mu$V in saline and approximately 9.4$\mu$V *in vivo* [Ye et al., 2024]. Moreover, in practice we expect voltage traces to be corrupted by currents from other nearby neurons, which our model treats as noise as well. Fixing the cell–probe distance at 10$\mu$m, we simulated 40 noisy EAPs at three increasing noise levels, ranging from $3\mu V$ to $30\mu V$. As expected, we found that higher noise leads to higher variance in the parameter estimates (fig. 2B). Nevertheless, estimates remained near the ground truth even at 30$\mu$V RMS, which represents a challenging, high-noise regime (fig. B.2).

We also examined the effect of increasing the distance between the cell and the recording site. Since extracellular voltage scales proportionally with $1/r$, the signal-to-noise ratio declined with distance. In line with this intuition, we found that larger distance between cell and recording site leads to higher variance in the estimate of membrane conductances (fig. B.1B).

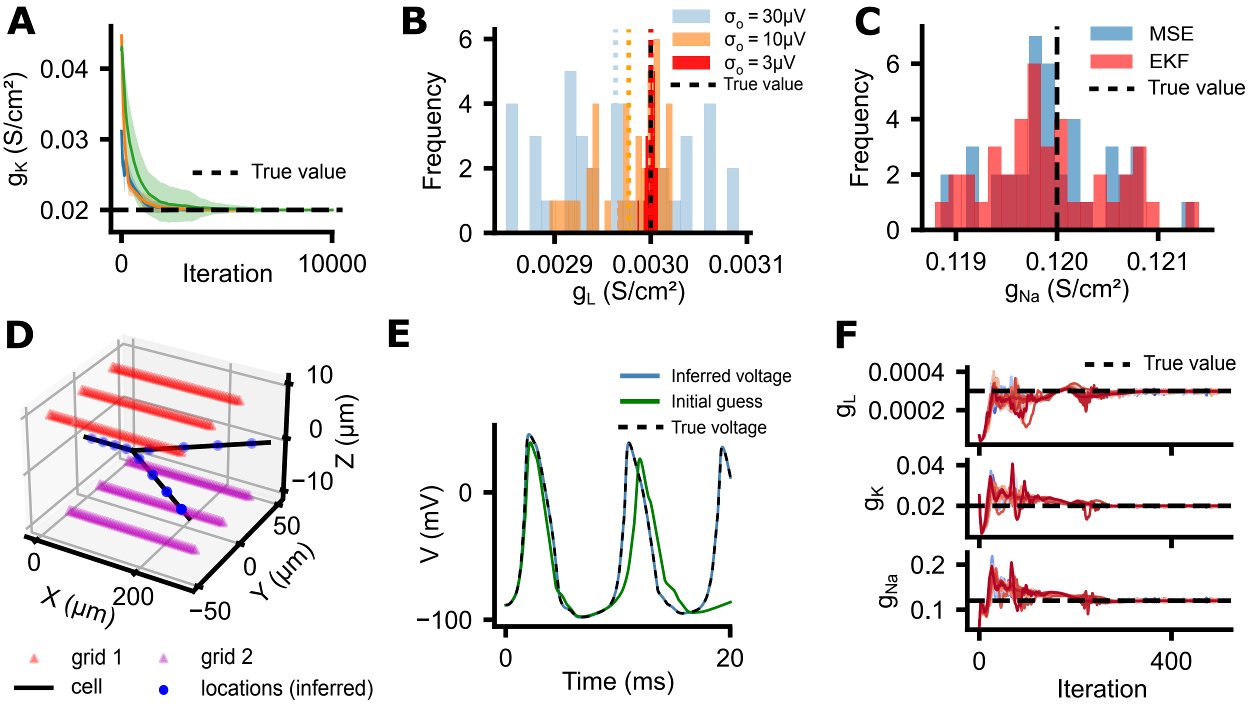

*Figure 2:* **Validation on simple morphologies.** Panels **A**–**C** show results for a single-compartment cell. **A**: Estimated $g_K$ over training iterations for three random initializations (mean +/- 1 sd). **B**: Distribution of inferred $g_L$ under different observation noise levels $\sigma_{obs}$ (colored dashed line indicate the mean). **C**: Distribution of inferred $g_{Na}$ for $\sigma_{obs} = 3\,\mu$V for MSE minimization and EKF. Panels **D**–**F** show results for a model with twelve compartments. **D**: A multi-compartment neuron and two sets of voltage recording grids (red and purple). The EKF correctly identifies the true location of the neuron (EKF in blue, true locations as black line). **E**: Recovery of membrane voltage in a compartment of the first branch, averaged over 40 datasets with $\sigma_{obs} = 1\,\mu$V. **F**: Inferred conductances $g_L$, $g_K$ and $g_{Na}$ of the first branch, across the 40 aforementioned datasets.

Finally, we compared our approach to a simpler alternative used in the original JAXLEY paper [Deistler et al., 2024]. Instead of maximizing the EKF marginal likelihood approximation, we directly minimized the mean squared error between the observed extracellular potentials and those predicted by a model with deterministic dynamics. Since the model is well-specified, we expect this simpler alternative to also perform well in this setting. Using the same setup (10 $\mu$m distance, 3 $\mu$V observation noise), we fit the same 40 traces using both methods. The resulting estimates were nearly indistinguishable (fig. 2C), suggesting that the local linearization used by EKF introduces negligible error. Additional figures can be found in Appendix B.1.

### 4.2 Validation on a multi-compartment neuron model

We next evaluated our method in a more realistic multi-compartment setting. We again assumed that the biophysical model is correctly specified and the dynamics are fully deterministic. Using JAXLEY, we simulated a synthetic neuron with three branches, each subdivided into four compartments, for a total of twelve compartments. Each compartment contained standard Hodgkin–Huxley ion channels, and the maximum conductances were allowed to vary independently across branches, yielding nine

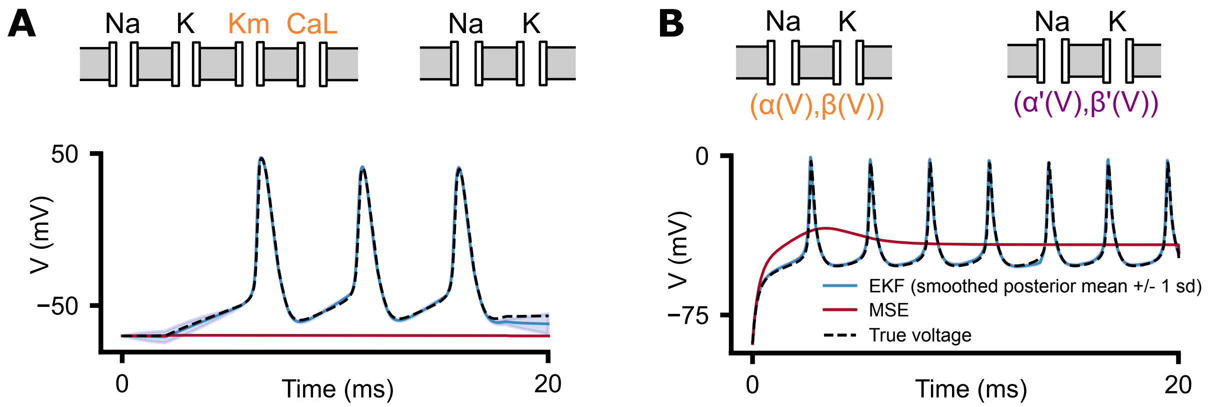

*Figure 3:* **Robustness to model misspecification.** Two types of channel misspecification are presented. **A**: missing channels (M-type $K^+$ and CaL). **B**: Incorrect gating dynamics for present channels. In both panels, recovered membrane voltage traces using MSE minimization and EKF are shown. EKF provides accurate voltage estimates despite misspecified channel models, whereas MSE minimization fails.

biophysical parameters to infer. The detailed experimental setup can be found in Appendix B.2. Unlike in the single-compartment case, we did not assume full knowledge of the cell's location: while we treated the number of branches, their connectivity, lengths, and radii to be known, the 3D spatial locations of the branch endpoints were treated as unknown. This introduced 18 additional geometric parameters, bringing the total number of parameters to 27.

A central question is whether these spatial locations are identifiable from extracellular recordings. If all recording sites lie in a single 2D plane—as is the case for a single Neuropixel probe—any mirror reflection of the neuron across that plane yields identical extracellular signals (formalization in Appendix C). To account for this unidentifiability, we simulated recordings from two separate probe positions, ensuring that the sets of recording sites did not lie in the same plane (fig. 2D, red and purple). We also ensured that the sites span the entire neuron to capture signal from all branches.

We fixed the observation noise to $1\mu$V and generated 40 sets of extracellular voltage recordings using this setup. To select an initialization, we first fixed a single set of recordings and sampled multiple random initializations from biologically plausible parameter ranges. We then chose the initialization that yielded the highest marginal likelihood after training. This initialization was held fixed and reused across all 40 simulated datasets. In this setup, our method consistently recovered the 3D locations of the center of the neuron's compartments (fig. 2D, blue vs black), the membrane voltage (fig. 2E), and the membrane conductances (fig. 2F), demonstrating the robustness and accuracy of biophysical and geometric inference in multi-compartment neuron models with unknown locations and membrane conductances. Additional figures are provided in Appendix B.2.

### 4.3 Robustness to model misspecification

In experimental settings, the true underlying biophysical dynamics of a neuron are rarely known with certainty. To evaluate the robustness of our approach under such model misspecification, we conducted experiments where the ground truth and inference models differ in their ion channel composition and dynamics. The detailed experimental setup and additional figures can be found in Appendix B.3.

**Omitted Ion Channels** In our first experiment, we generated synthetic data from a single-compartment neuron model containing both classical Hodgkin–Huxley (HH) channels and two additional conductances: an M-type potassium current and a calcium current. During inference, we deliberately used a simplified model containing only the HH channels, omitting the additional ones (fig. 3A).

Since we allow for dynamics noise in our model with process noise, we were able to accurately recover the underlying membrane voltage, despite this substantial misspecification. In contrast, naive fitting via mean squared error (MSE) minimization failed to capture the dynamics, yielding voltage estimates that diverged substantially from the ground truth. This result highlights the importance of allowing for uncertainty in the state evolution, using our EKF-based approach.

**Perturbed Gating Dynamics** We next evaluated the effect of incorrect gating kinetics. Here, we retained the correct channel types in the inference model but perturbed the rate constants $\alpha(V)$ and $\beta(V)$ used in gating variable dynamics, simulating errors in channel parameter specification (fig. 3B). Once again, EKF-based inference provided significantly more accurate estimates of both membrane voltage and channel states compared to MSE minimization. These results suggest that our approach can tolerate moderate misspecification in gating dynamics. Together, these experiments demonstrate that EKF-based inference, combined with stochastic modeling of dynamics, confers robustness to common forms of model misspecification.

## 5 Application to real morphologies

Having established the method's efficiency in controlled settings, we next apply it to morphologically detailed neuron models derived from real reconstructions, including a retinal ganglion cell (RGC) and a CA1 pyramidal neuron.

### 5.1 Application to 2D retinal ganglion cell

We applied our method to a morphologically detailed model of a retinal ganglion cell (RGC), replicating conditions typical of *in vitro* studies using planar multi-electrode arrays (MEAs) [Vilkhu et al., 2024]. In these setups, retinal tissue is placed ganglion-side down on the MEA, enabling simultaneous current injection and extracellular voltage recording from hundreds of electrodes (fig. 4A). We adopted a reconstructed RGC morphology from Vilkhu et al. [2024], consisting of a soma and a complex dendritic arbor discretized into 122 compartments. The membrane model includes modified Hodgkin–Huxley sodium and potassium channels with non-standard kinetics. Following Vilkhu et al. [2024], we assumed shared maximum conductances across all dendritic compartments, yielding a total of six parameters to estimate. For details of the model, see Appendix D.

We here assumed the spatial location of the neuron to be known and constrained to a 2D plane, as is typical in retinal MEA experiments [Madugula et al., 2022, Sekirnjak et al., 2006, Vilkhu et al., 2024]. The cell was stimulated via somatic current injection, and extracellular voltages were recorded using a simulated MEA with 30$\mu$m inter-electrode spacing, matching the geometry of the original 512-electrode array. To reduce computational cost while maintaining full spatial coverage, we used a subset of 169 electrodes spanning the entire neuron. For each noise level (0.1$\mu$V and 10$\mu$V), we simulated ten extracellular action potentials (EAPs, fig. 4B).

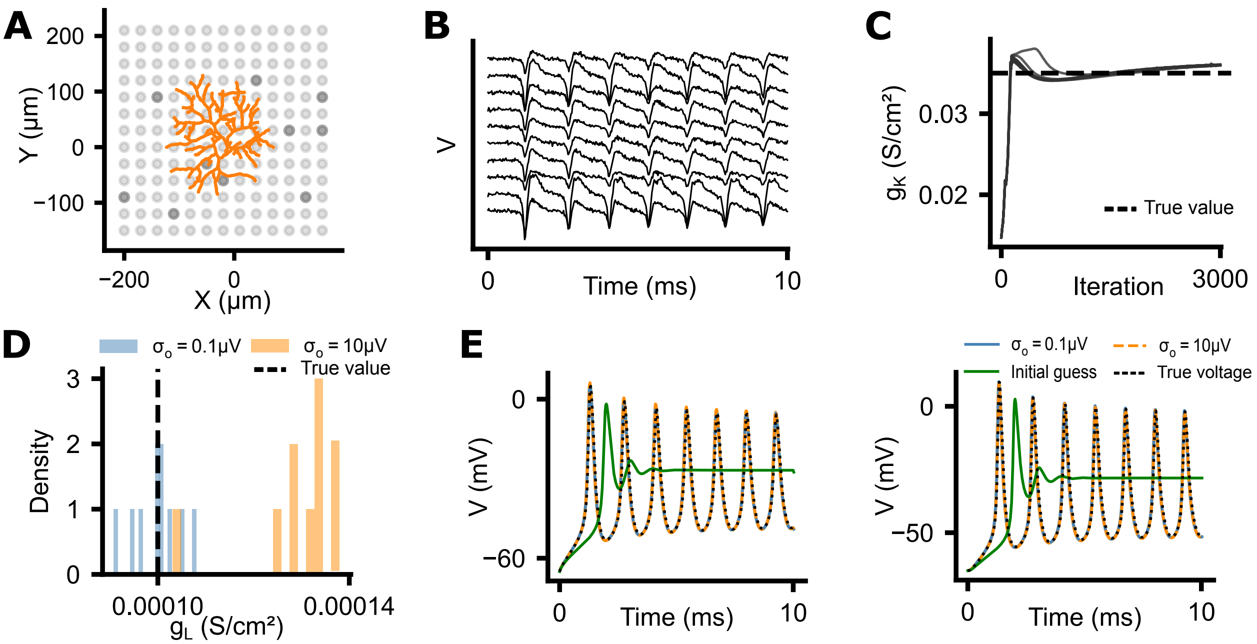

*Figure 4:* **Inference of retinal ganglion cell (RGC) properties given multi-electrode array (MEA) recordings.** **A**: RGC cell geometry and electrode layout. Darker shaded MEA sites match the voltage traces shown in panel B. **B**: Simulated extracellular recordings at ten sites for noise level $\sigma_{\text{obs}} = 10\,\mu$V. **C**: Estimation of $g_K$ in the dendrites for 10 optimization runs under noise level $\sigma_{\text{obs}} = 0.1\mu$V. **D**: Distribution of inferred $g_L$ for $\sigma_{\text{obs}} \in \{0.1, 10\}\,\mu$V. EKF identifies true parameters for sufficiently low values of noise. **E**: Recovered membrane voltage in the soma (left) and in a distal dendritic compartment (right) for both noise levels (mean +/- 1 sd). EKF matches the true voltage dynamics.

Given the high dimensionality of the latent state space ($d = 610$), we used our diagonal EKF for efficient inference. We used multiple random initializations and selected the one that yielded the highest marginal likelihood. Our method accurately recovered the underlying conductances, exhibiting only minor bias (fig. 4C). While this bias increased with higher observation noise (fig. 4D), performance remained robust even at noise levels typical of MEA recordings (e.g., 10$\mu$V [Multi Channel Systems MCS GmbH, 2020]). Notably, the inferred model was still able to faithfully reconstruct the true membrane voltage trace, highlighting the reliability of the approach under realistic noise conditions (fig. 4E). Additional figures are provided in Appendix D.

### 5.2 Application to CA1 hippocampal pyramidal cell

Finally, we applied our approach to a morphologically detailed model of a rat CA1 hippocampal pyramidal neuron, based on the C10 model introduced by Cutsuridis et al. [2010]. This model has been used in prior computational studies to examine hippocampal circuit function and memory encoding, capturing the integration and propagation of signals across dendritic and axonal compartments [Cutsuridis et al., 2010, Cutsuridis and Taxidis, 2013]. We recreated the full morphology of the C10 cell, including the soma, axon, basal dendrites, and apical dendrites (fig. 5A). The cell was equipped with region-specific Hodgkin–Huxley-type channels whose conductances and gating dynamics varied by subcellular location [Cutsuridis et al., 2010, Poirazi et al., 2003b, Migliore et al., 1999]. This introduced 24 distinct maximum

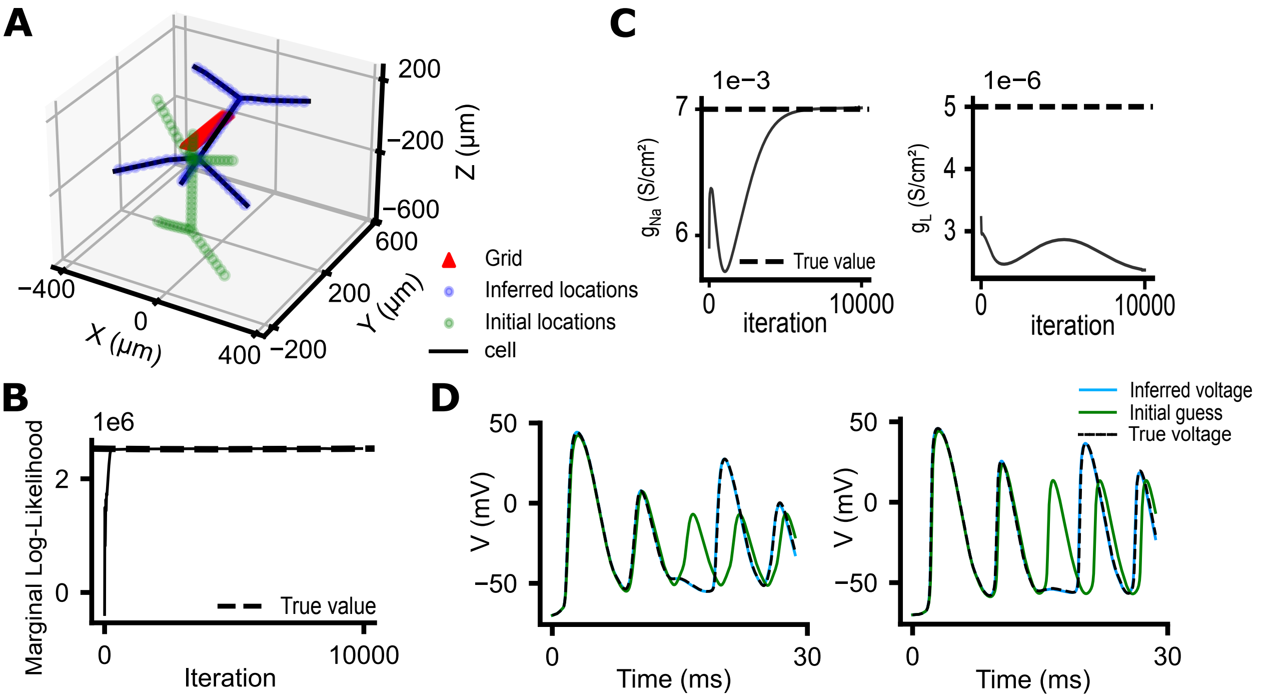

*Figure 5:* **Inference of membrane properties and locations of a C10 model of a pyramidal cell.** **A**: Pyramidal cell placed near a Neuropixel Ultra probe. Estimated compartment locations closely match true cell position. **B**: Marginal log-likelihood during optimization, converging to the ground truth value. **C**: Inferred conductances $g_{\mathrm{Na}}$ and $g_L$ in selected dendritic compartments. **D**: Recovered membrane voltage traces in dendrites of the stratum radiatum (left) and lacunosum-moleculare (right).

conductances to infer. The neuron was stimulated via somatic current injection, and extracellular traces were recorded using a simulated Neuropixel Ultra probe. Our virtual probe matched the true probe's geometry, including the number of measurement sites and inter-site spacing.

We treated the spatial location of the neuron as unknown up to a rigid-body transformation, adding six parameters for rotation and translation and bringing the total number of parameters to 30. We initialized conductances with biologically plausible values for CA1 neurons. The detailed experimental setup and additional figures are provided in Appendix E. Following Carnevale and Hines [2006], the cell was discretized into 60 compartments, resulting in a latent state of dimension 360. Since this model size remained computationally tractable, we ran the full EKF rather than using the diagonal approximation. To avoid being trapped in a local maximum, we ran 100 independent restarts and retained the solution with the highest final marginal log-likelihood (fig. 5B).

Our method successfully recovers the neuron's spatial configuration by estimating the 3D locations of compartment centers (fig. 5A, black vs. blue), and accurately reconstructs membrane voltage (fig. 5D). It also recovers some of the maximum conductances, though not all (fig. 5C). This outcome is consistent with a well-documented phenomenon in conductance-based neuron models: parameter degeneracy, where multiple combinations of channel conductances can yield virtually indistinguishable voltage dynamics [Prinz et al., 2004, Sterratt et al., 2023]. Despite this, our method reliably recovers the neuron's functional dynamics and spatial structure, demonstrating that biophysical and geometric inference from high-density extracellular recordings is feasible in realistic, large-scale neuron models.

### 6 Conclusions, Limitations, and Future Work

**Summary** We presented an approach for identifying detailed biophysical neuron models from high-density extracellular voltage recordings. Our method recovers not only membrane conductances but also the spatial position of a neuron relative to the recording probe, solely from extracellular data. We presented an approach to state inference and parameter estimation using an Extended Kalman Filter (EKF), which provides robustness to model misspecification and accurate inference even when the assumed dynamics deviate from the true underlying system.

**Limitations and Future Work** One limitation of using an EKF to estimate model parameters is its computational cost: while we have developed a diagonal approximation to the filtering distribution, running an EKF is still computationally more costly than MSE minimization, especially for very large models. On top of this, any approximation made by the EKF (linearizing the dynamics, diagonal approximation) could bias parameter recovery. While we empirically found that our method could accurately recover parameters across a range of tasks, the approximations might have stronger effects on other models. Moreover it is common for biophysical models to exhibit parameter degeneracy: different combinations of maximum conductances can produce similar voltage dynamics, which can limit the identifiability of individual biophysical parameters, even when predictions match the data well.

Finally, while our results demonstrate the feasibility of biophysical inference from extracellular recordings at the single-cell level in idealized settings, extending this framework to real *in vivo* conditions remains a significant challenge. In particular, real recordings involve complex networks of interacting neurons. A natural next step is to move beyond isolated cells and model local neural populations surrounding the probe. Given knowledge of the targeted brain region, one could incorporate priors on network structure and cell types informed by anatomical and physiological studies, enabling more comprehensive modeling of *in vivo* extracellular signals.

**Conclusion** Overall, our method—based on a combination of state-space modeling and differentiable simulation—opens up possibilities to estimate properties across the entire morphologies of neurons from extracellular signals alone. This approach will enable new analysis of how cells generate action potentials and open new avenues for studying the biophysical contributions to neural computation.

### Acknowledgments

We thank Alisa Levin, Noah Cowan, and other members of the Linderman Lab for helpful feedback throughout this project. We are also grateful to Amrith Lotlikar, Michael Sommeling, and E.J. Chichilnisky for sharing valuable insights into retinal ganglion cell physiology and model fitting approaches. This work was supported by grants from the NSF EFRI BRAID Program (2223827), the NIH BRAIN Initiative (U19NS113201, R01NS131987, R01NS113119, & RF1MH133778), the NSF/NIH CRCNS Program (R01NS130789), and the Sloan, Simons, and McKnight Foundations. It was also supported by the German Research Foundation (DFG) through Germany's Excellence Strategy (EXC 2064 – Project number 390727645) and the CRC 1233 "Robust Vision", and the European Union (ERC, "DeepCoMechTome", ref.101089288).

### A Resources

All simulations and inference experiments were conducted using four open-source Python libraries:

- JAX (version 0.4.31, Apache License 2.0): A high-performance numerical computing library based on XLA, providing composable function transformations and GPU/TPU support. Available at: https://github.com/google/jax.
- JAXLEY [Deistler et al., 2024] (version 0.7.0, Apache License 2.0): A differentiable simulator for multi-compartment Hodgkin–Huxley models, supporting GPU acceleration, just-in-time compilation, and gradient-based parameter optimization. Available at: https://github.com/jaxleyverse/jaxley.
- DYNAMAX [Linderman et al., 2025] (version 0.1.4, MIT License): A library for state-space modeling and inference, used here for implementing the Extended Kalman Filter (EKF) and its diagonal approximation. Available at: https://github.com/probml/dynamax.
- SPARSEJAC [Schubert, 2023] (version 0.2.0, MIT License): A library for efficient sparse Jacobian computation using graph coloring and automatic differentiation. Available at: https://github.com/mfschubert/sparsejac.

For experiments on large-scale neuron models (e.g., the retinal ganglion cell and C10 model of a pyramidal neuron), we used an NVIDIA H100 GPU. All other experiments were run on a MacBook Pro with an Apple M1 Pro (8-core CPU, 16 GB RAM) running macOS 13.4.

### B Additional Details and Figures for Section 4

This section provides additional experimental details, biophysical parameters, and figures related to the validation experiments presented in Section 4.

The extracellular resistivity was assumed to be homogeneous and equal to $300\,\Omega \cdot \text{cm}$, and the specific membrane capacitance was fixed to $C_m = 1\,\mu\text{F/cm}^2$ across all compartments. While we assume homogeneous extracellular resistivity, this simplification neglects known spatial variability [Gold et al., 2006], which could be addressed in future work. Extracellular voltages were recorded at a sampling rate of 40 kHz.

All voltages and channel states were initialized at their steady-state values at rest, and thus we assumed the initial state to be known in the EKF. We set the initial covariance matrix of the EKF to $\Sigma_0 = 0.1I$. Observation noise was also assumed to be known, as commercial devices that record extracellular potentials typically provide noise specifications [Ye et al., 2024, Multi Channel Systems MCS GmbH, 2020].

In Sections 4.1 and 4.2, where the state dynamics were well-specified and deterministic, we fixed the standard deviation of the membrane potentials and channel states to small values: $\sigma_v = 0.0001\,\text{mV/ms}^{1/2}$, and $\sigma_{\text{state}} = 0.00001\ \text{ms}^{-1/2}$ (same for all the channels) for all compartments. These values ensured numerical stability while avoiding degenerate gradients during optimization.

| | Na | K | Leak |
|---|---|---|---|
| Maximum conductance ($S/cm^2$) | 0.12 | 0.02 | 0.003 |
| Reversal potential (mV) | 53.0 | −107.0 | −88.5188 |

*Table 1:* Biophysical parameters of the single-compartment neuron model.

| | Na | K | Leak |
|---|---|---|---|
| Reversal potential (mV) | 53.0 | −107.0 | −88.5188 |

*Table 2:* Reversal potentials used in the multi-branch neuron model.

#### B.1 Validation on Single-Compartment Neuron Model

We evaluated a single-compartment neuron model as a baseline for benchmarking. The model consists of a cylindrical compartment of length 24 $\mu$m and radius 12 $\mu$m, equipped with classical Hodgkin–Huxley (HH) sodium, potassium, and leak channels [Hodgkin and Huxley, 1952]. The maximum conductances and reversal potentials are provided in Table 1. A constant current of amplitude 2.5 nA was injected for 20 ms.

As the neuron–electrode distance increased, the signal-to-noise ratio (SNR) decreased, resulting in higher bias and variance in inferred conductances (fig. B.1B). At short distances with high SNR, both MSE minimization and EKF yielded comparable estimates, indicating that EKF's linearization introduced minimal error (fig. B.1C). As observation noise increased, estimation accuracy declined (fig. B.1A), but even at 30 $\mu$V RMS—a challenging noise regime (fig. B.2C)—estimates remained close to the ground truth. Notably, the true voltage remained within two standard deviations of the mean of the reconstructed voltage traces for most of the signal (fig. B.3), indicating robustness even under challenging conditions.

#### B.2 Validation on Multi-Compartment Neuron Model

We also evaluated a multi-branch neuron model to test conductance and geometry inference. The model consists of three branches, each discretized into four cylindrical compartments of radius 2 $\mu$m, for a total of 12 compartments. Each compartment contains classical Hodgkin-Huxley sodium, potassium, and leak channels [Hodgkin and Huxley, 1952]. Reversal potentials are shared across all branches (Table 2), while conductances and lengths vary by branch (Table 3). A current of 0.5 nA was injected in the first compartment of branch 1 (soma) for 20 ms.

All the maximum conductances were inferred accurately for all 40 datasets simulated with $\sigma_{obs} = 1\mu$V (fig. B.4).

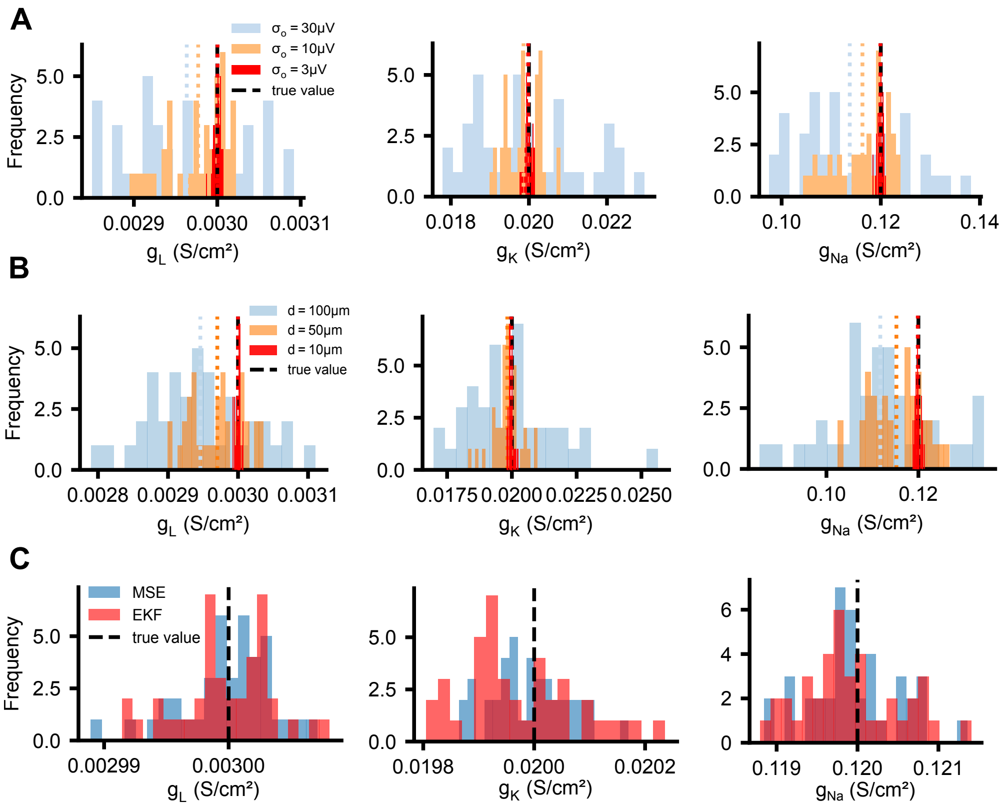

*Figure B.1:* **Validation on synthetic single-compartment. A**: Distributions of inferred maximum conductances under different observation noise levels $\sigma_{\text{obs}}$ (colored dashed lines indicate the means). **B**: Distributions of inferred maximum conductances for different cell-probe distances (colored dashed lines indicate the means). **C**: Distributions of inferred maximum conductances for MSE minimization and EKF, with $\sigma_{\text{obs}} = 3\,\mu$V and $d = 10\mu$m.

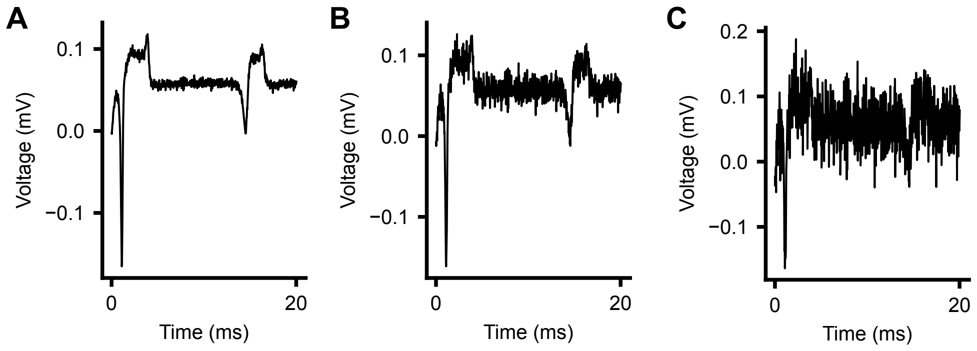

*Figure B.2:* **Extracellular voltage traces simulated for different noise levels in the single-compartment model. A**: $\sigma_{obs} = 3\mu$V, **B**: $\sigma_{obs} = 10\mu$V, **C**: $\sigma_{obs} = 30\mu$V represents a challenging noise regime. The electrode is $10\mu$m away from the cell.

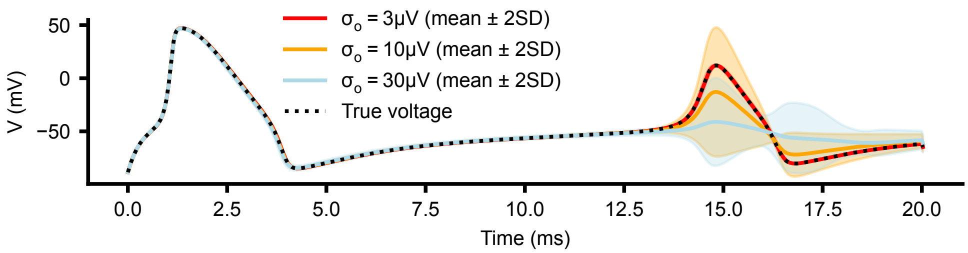

*Figure B.3:* **Recovery of membrane voltage for different observation noise levels in the single-compartment model.** The mean and standard deviation are estimated by considering 40 datasets simulated at fixed observation noise. Even in challenging noise regimes ($\sigma_{obs} = 30\mu$V), the true voltage remains within two standard deviations of the mean of the reconstructed voltage traces for most of the signal.

| **Branch** | $g_{\text{Na}}$ | $g_{\text{K}}$ | $g_{\text{Leak}}$ | Length |
|---|---|---|---|---|
| 1 | 0.12 | 0.02 | 0.0003 | 24.0 |
| 2 | 0.08 | 0.03 | 0.0004 | 50.0 |
| 3 | 0.10 | 0.008 | 0.0001 | 50.0 |

*Table 3:* Maximum conductances (in $\text{S/cm}^2$) and branch lengths (in $\mu$m) in the multi-branch neuron model.

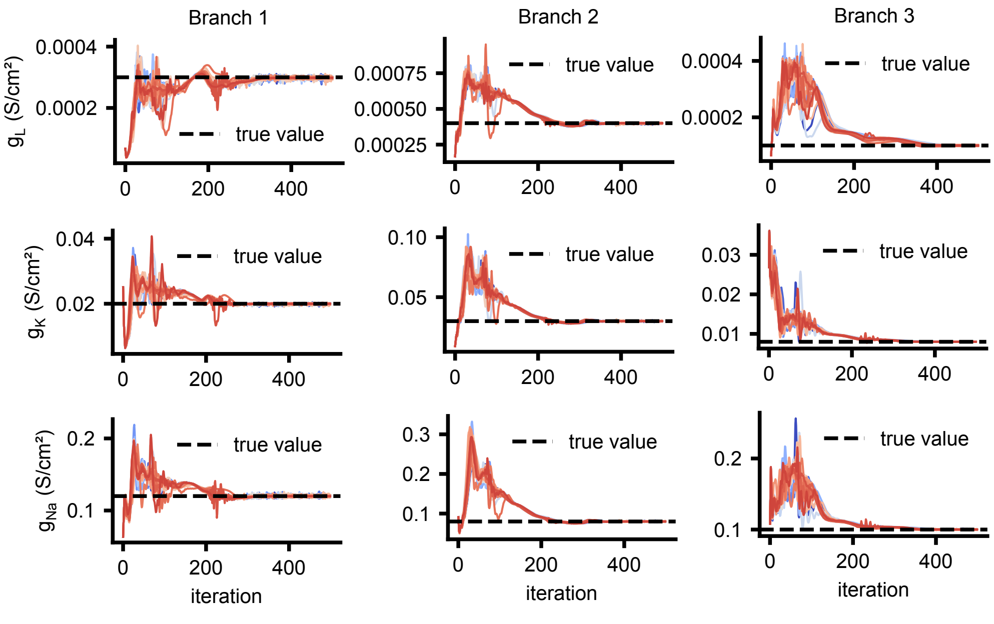

*Figure B.4:* **Validation on synthetic multi-branch neuron.** Inferred maximum conductances $g_{\text{L}}$, $g_{\text{K}}$ and $g_{\text{Na}}$ for all the branches, across 40 datasets simulated with $\sigma_{\text{obs}} = 1\,\mu$V.

### B.3 Robustness to Model Misspecification

In the main text, we considered two forms of model misspecification: missing ion channels and incorrect gating kinetics.

| Sodium ($Na^+$) | Potassium ($K^+$) |
|---|---|
| $\alpha_m = \dfrac{0.32(V+47)}{1-e^{-(V+47)/4}}$ | $\alpha_n = \dfrac{0.032(V+45)}{1-e^{-(V+45)/5}}$ |
| $\beta_m = \dfrac{0.28(V+20)}{e^{(V+20)/5}-1}$ | $\beta_n = 0.5\, e^{-(V+50)/40}$ |
| $\alpha_h = 0.128\, e^{-(V+43)/18}$ | |
| $\beta_h = \dfrac{4}{1+e^{-(V+20)/5}}$ | |

*Table 4:* Rate functions for sodium ($Na^+$) and potassium ($K^+$) channels used in the single-compartment model for the missing channel experiment.

| M-type $K^+$ (KM) | L-type $Ca^{2+}$ (CaL) |
|---|---|
| $p_\infty = \dfrac{1}{1+e^{-0.1(V+35)}}$ | $\alpha_q = 0.055\dfrac{V+27}{1-e^{-(V+27)/3.8}}$ |
| $\tau_p = \dfrac{4000}{3.3\, e^{0.05(V+35)} + e^{-0.05(V+35)}}$ | $\beta_q = 0.94\, e^{-(V+75)/17}$ |
| | $\alpha_r = 0.000457\, e^{-(V+13)/50}$ |
| | $\beta_r = \dfrac{0.0065}{1+e^{-(V+15)/28}}$ |

*Table 5:* Rate functions for M-type potassium (KM) and L-type calcium (CaL) channels used in the single-compartment model for the missing channel experiment.

**Omitted Ion Channels.** To test robustness to missing channels, we simulated data from a single-compartment model (length 10$\mu$m, radius 1$\mu$m) containing M-type potassium (KM) and L-type calcium (CaL) channels (Table 5) in addition to Hodgkin-Huxley (HH) channels (Table 4). Inference was performed using a model containing only the HH channels. A step current of 0.004 nA was injected for 20 ms. Extracellular voltages were recorded at a single electrode located 10 $\mu$m away from the soma, with a noise level of $\sigma_{obs} = 0.1\,\mu$V. Biophysical parameters for the simulation model are shown in Table 6.

During EKF inference, we fixed the standard deviation of the channel states to $\sigma_{\text{state}} = 0.00001$ ms$^{-1/2}$ (same for all the channels) and treated $\sigma_v$ as a free parameter to capture voltage uncertainty due to model mismatch. We performed hyperparameter sweeps over learning rates and initial values of the parameters, selecting the configuration with the highest marginal log-likelihood.

For MSE-based optimization, we used Adam and reported the result with the lowest final MSE.

**Perturbed Gating Dynamics.** To evaluate robustness to incorrect kinetics, we generated data using gating functions from a retinal ganglion cell (RGC) model (Table 8) and performed inference using the original Hodgkin-Huxley kinetics [Hodgkin and Huxley, 1952]. The neuron was a single cylindrical cable (length 24 $\mu$m, radius 2 $\mu$m), discretized into four compartments. A constant current of 0.5 nA

| | Na | K | Leak | KM | CaL |
|---|---|---|---|---|---|
| Maximum conductance (S/cm$^2$) | 0.05 | 0.005 | 0.0001 | 0.000004 | 0.0001 |
| Reversal potential (mV) | 50.0 | −90.0 | −70.0 | −90.0 | 120 |

*Table 6:* Biophysical parameters for the single-compartment neuron model used in the missing channel experiment.

| | Na | K | Leak |
|---|---|---|---|
| Maximum conductance (S/cm$^2$) | 0.12 | 0.02 | 0.003 |
| Reversal potential (mV) | 53.0 | −107.0 | −88.5188 |

*Table 7:* Biophysical parameters for the single-branch neuron model used for the kinetic mismatch experiment.

was injected into the first compartment (soma) for 20 ms. Extracellular voltages were recorded at 10 different locations with noise level $\sigma_{obs} = 0.1\,\mu$V. Biophysical parameters for the simulation model are shown in Table 7.

During EKF inference, we treated the voltage standard deviation $\sigma_v$ and channel noise levels $\sigma_{\mathrm{K}}, \sigma_{\mathrm{Na}}, \sigma_{\mathrm{Leak}}$ as learnable parameters. While only the sodium and potassium kinetics are misspecified, we allowed separate noise levels for each channel type to provide flexibility in how uncertainty is accounted for. We also used the same $\sigma_v, \sigma_{\mathrm{K}}, \sigma_{\mathrm{Na}}, \sigma_{\mathrm{Leak}}$ across all compartments, though future work could consider compartment-specific noise parameters. As before, we performed grid search over learning rates and initializations, selecting the result with the highest marginal log-likelihood.

For MSE optimization, we used Adam and selected the result with the lowest MSE.

The EKF smoothed posterior mean of the voltage recovers the true voltage trace in each of the four compartments, whereas MSE minimization fails (fig. B.5).

### C Unidentifiability of 3D compartment locations

In this section, we show that when inferring a neuron's spatial position from extracellular recordings, the geometry of the recording sites can impose inherent unidentifiability in the recovered compartment locations.

Our multi-compartment model represents the neuron as a collection of compartments, each treated as a point source of transmembrane current located at its center. Hence, estimating a neuron's spatial configuration reduces to estimating the 3D positions of its compartments. According to Eq. 3, the extracellular potential measured at a given electrode depends only on the transmembrane currents and the inverse distance to each compartment. Therefore, if the distances between each compartment and all electrodes remain unchanged, the resulting extracellular signals will be identical.

We first consider the case where all electrodes lie on a single line (fig. C.1A). Fix a single compartment

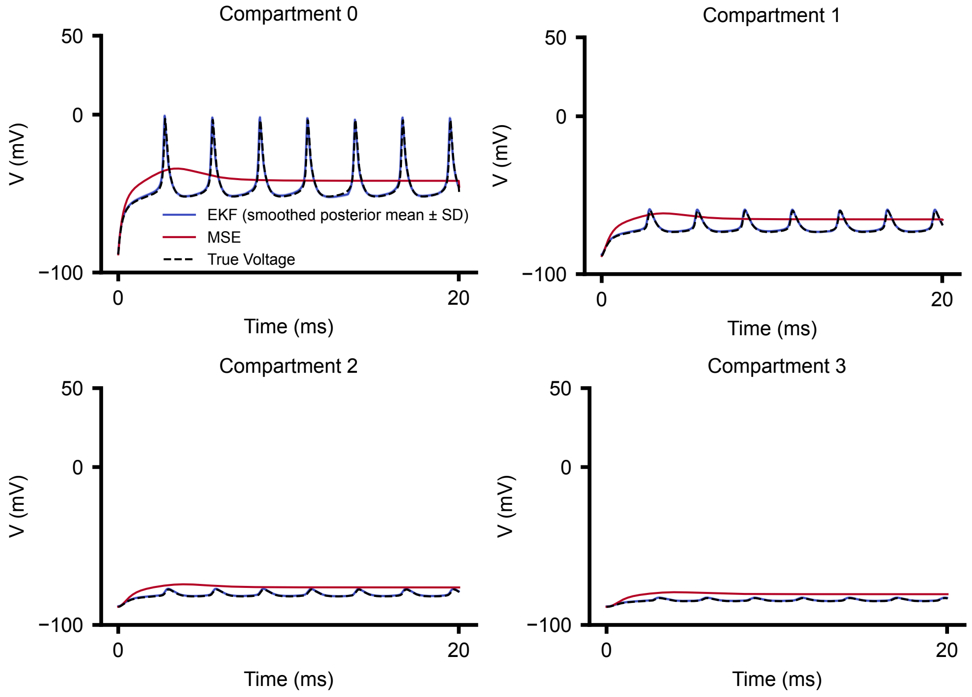

*Figure B.5:* **Robustness to misspecified channel kinetics.** The EKF smoothed posterior mean of the voltage recovers the true voltage trace in each of the four compartments, whereas MSE minimization fails.

at some position in 3D space (black). The set of all locations that preserve its distance to a given site is a sphere. The intersection of these spheres—corresponding to multiple sites aligned on a line—is a circle. Thus, any position on this circle (blue dashed circle) yields the same set of distances (and hence the same extracellular potentials), rendering the compartment's position unidentifiable up to a rotation around the axis defined by the recording sites.

Next, suppose the electrodes lie on two parallel lines, forming a planar grid (fig. C.1B). In this case, for a given compartment (black), the set of equidistant locations from the first line of sites lies on a circle (blue), and similarly for the second line (green). The only positions that simultaneously preserve distances to both sets of sites lie at the intersection of these two circles. This intersection consists of exactly two points: the true location of the compartment and its mirror reflection across the plane defined by the electrodes (grey).

This ambiguity persists even with more than two lines of electrodes, as long as they all lie in the same plane. For example, fig. C.1C shows three parallel lines of recording sites in a planar grid, viewed from above. The mirror-reflected location remains the only position (besides the true one) that produces identical distances to all recording sites.

In general, if all electrodes lie in a 2D plane (e.g., $z = 0$), then for any compartment, the reflection of its true location across this plane yields the same distances to all electrodes. Consequently, the extracellular voltages at all sites are unchanged. This holds independently of the number or spacing of electrodes and applies to all compartments of a multi-compartment model. Therefore, in the absence of additional

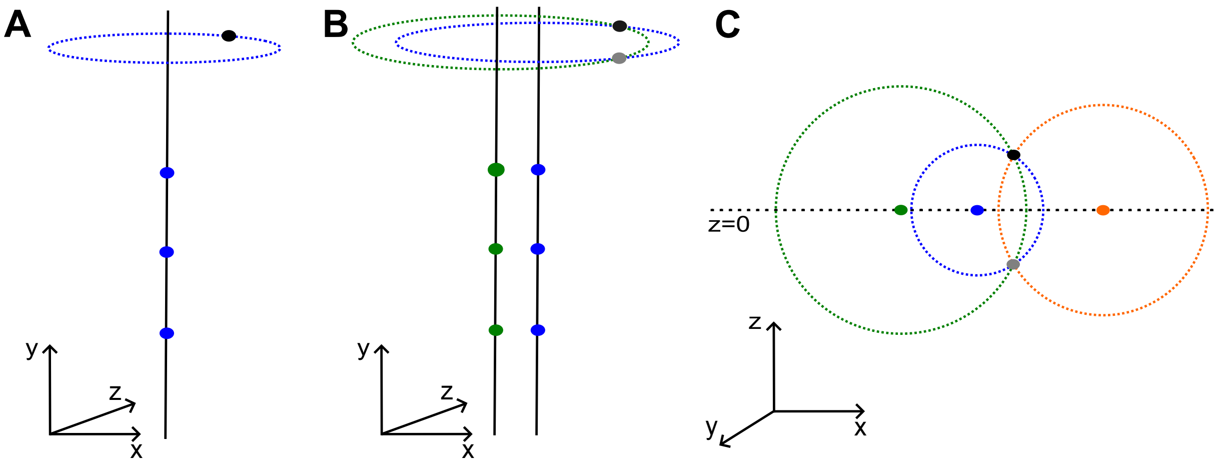

*Figure C.1:* **Unidentifiability of the positions.** **A**: The distance between the compartment (black) and each electrode (blue) is unchanged as it moves along the blue dashed circle. It follows that any position of the compartment on the circle yields the same observations at all the electrodes. **B**: Compartment's distance to each blue (resp. green) electrode is unchanged as it moves along the blue (resp. green) dashed circle. Hence, the grey location is the only other position that yields the same observations as the black one at all electrodes. **C**: Panel **B** viewed from above with now *three* parallel lines containing electrodes (green, blue, orange ones). Since all the electrodes lie in a 2D-plane ($z = 0$), the only position that yields the same observations as the true location at each electrode is the true location's reflection with respect to the plane $z = 0$.

constraints, the entire neuron morphology is identifiable only up to reflections of its compartments across the recording plane.

This ambiguity can be partially mitigated by incorporating structural constraints or prior knowledge about the neuron. For example, if the cell is known to consist of a single linear branch, then the configuration of all compartments is restricted to lie along a line. In this case, the full morphology is only ambiguous up to a reflection of that line across the recording plane—substantially reducing the space of indistinguishable configurations. Similarly, if the compartmental connectivity is known (i.e., which compartments are connected to each other and in what topology), then only configurations that preserve both the pairwise distances to electrodes and the known connectivity are allowed. While this does not fully resolve the mirror symmetry, it rules out arbitrary spatial rearrangements. Finally, full identifiability can be achieved by breaking the planar symmetry of the recording sites. For example, recordings from two probes placed at different depths or orientations—such that their electrodes do not lie in the same plane—remove the reflection ambiguity and enable unique localization of each compartment in 3D space.

## D Retinal Ganglion Cell (RGC) Model

We use a morphologically detailed model of a retinal ganglion cell (RGC), based on a reconstruction from Vilkhu et al. [2024]. The morphology was originally provided as a NEURON `.hoc` file,[1] which we converted to SWC format for compatibility with JAXLEY. The model includes the full soma and dendritic arbor, and was discretized into 122 compartments, assigning one compartment per branch.

[1] https://github.com/ramanvilkhu/rgc_simulation_multielectrode

The ion channel model consists of Hodgkin–Huxley (HH) conductances: fast sodium, delayed-rectifier potassium, and leak channels. Voltage-dependent rate constants are listed in Table 8, and channel reversal potentials in Table 9. Region-specific maximum conductances for soma and dendrites are given in Table 10. All values were taken directly from Vilkhu et al. [2024], with a minor adjustment to the leak reversal potential to ensure resting membrane potentials near −65 mV across compartments. Note that Vilkhu et al. [2024] also consider two additional channels (a calcium channel and a calcium-gated potassium channel), which we neglected in this experiment. Including these channels could be the subject of future work.

The axial resistivity was 143.2 $\Omega \cdot$ cm, the membrane capacitance was 1 $\mu$F/cm$^2$ across all compartments, and the extracellular resistivity was 1000 $\Omega \cdot$ cm.

To generate synthetic extracellular recordings, we applied a constant somatic current injection of 1.0 nA for 10 ms. Voltage recordings were simulated using a synthetic planar multi-electrode array (MEA) with a sampling rate of 40 kHz.

We fit this model using our diagEKF implementation. All voltages and channel states were initialized at their steady-state values at rest, and thus we assumed the initial state to be known in diagEKF. We set the initial covariance matrix to $\Sigma_0 = 0.1I$. Observation noise was also assumed to be known, as commercial devices that record extracellular potentials typically provide noise specifications [Ye et al., 2024, Multi Channel Systems MCS GmbH, 2020]. Since the state dynamics were well-specified and deterministic, we fixed the standard deviation of the membrane potentials and channel states to small values: $\sigma_v = 0.001\,\text{mV/ms}^{1/2}$, and $\sigma_{\text{state}} = 0.0001\ \text{ms}^{-1/2}$ (same for all the channels) for all compartments. These values ensured numerical stability while avoiding degenerate gradients during optimization.

We optimized conductance parameters using the Adam optimizer. We tried multiple learning rates and initialized values uniformly at random within the bounds shown in Table 11. We ran 100 random initializations with different seeds and selected the result that achieved the highest marginal log-likelihood.

All maximum conductances were accurately inferred across all 10 datasets simulated at a fixed noise level of $\sigma_{\text{obs}} = 0.1\,\mu$V, with only minor biases observed for a few conductances (fig. D.1). As expected, increasing the observation noise to $\sigma_{\text{obs}} = 10\,\mu$V generally led to higher bias and variance of the estimates, though the inferred values remained close to the ground truth (fig. D.2). In both noise conditions, membrane voltage was accurately recovered throughout the cell (fig. D.3), and the marginal log-likelihood converged to its optimal value (fig. D.4).

## E CA1 Pyramidal Neuron (C10 Model)

We use the C10 model of a CA1 pyramidal neuron, originally developed by Cutsuridis et al. [2010]. The morphology is provided as a NEURON `.hoc` file,[2], which we converted to SWC format for compatibility with JAXLEY. This file specifies the full 3D morphology, including the diameters and lengths of all the branches (Table 14). As shown in fig. E.1, the neuron is divided into 15 anatomical sections.

Following Cutsuridis et al. [2010], we applied the compartmentalization heuristic of Carnevale and

[2] https://github.com/tomko-neuron/HippoUnit/tree/master/Cutsuridis_CA1_2010

| **Sodium ($Na^+$)** | **Potassium ($K^+$)** |
|---|---|
| $\alpha_m = \dfrac{2.725(V+35)}{1-e^{-(V+35)/10}}$ | $\alpha_n = \dfrac{0.09575(V+37)}{1-e^{-(V+37)/10}}$ |
| $\beta_m = 90.83\, e^{-(V+60)/20}$ | $\beta_n = 1.915\, e^{-(V+47)/80}$ |
| $\alpha_h = 1.817\, e^{-(V+52)/20}$ | |
| $\beta_h = \dfrac{27.25}{1+e^{-(V+22)/10}}$ | |

*Table 8:* Rate functions in the RGC model.

| | Na | K | Leak |
|---|---|---|---|
| Reversal potential (mV) | 60.60 | −101.34 | −67.1469 |

*Table 9:* Reversal potential for ion channels in the RGC model.

| | $g_{\text{Na}}$ | $g_{\text{K}}$ | $g_{\text{Leak}}$ |
|---|---|---|---|
| Dendrites | 0.06 | 0.035 | 0.0001 |
| Soma | 0.06 | 0.035 | 0.0001 |

*Table 10:* Maximum conductances in the RGC model (in $\text{S/cm}^2$).

| **Region** | $g_{\text{L}}$ | $g_{\text{Na}}$ | $g_{\text{K}}$ |
|---|---|---|---|
| Soma | [0.0001, 0.005] | [0.05, 0.1] | [0.01, 0.05] |
| Dendrite | [0.0001, 0.005] | [0.05, 0.1] | [0.01, 0.05] |

*Table 11:* Initialization bounds for the maximum conductances in the RGC model (in $\text{S/cm}^2$).

Hines [2006] to determine the number of compartments per section. Specifically, a section of length $L$ is discretized into

$$N = \left\lfloor \frac{1}{2}\left(\frac{L}{0.1 \cdot \lambda_f(100)} + 0.9\right)\right\rfloor \cdot 2 + 1,$$

where the frequency-dependent electrotonic length is defined as $\lambda_f(f) = \frac{1}{2}\sqrt{\frac{d}{\pi f R_a C_m}}$, with $d$ the diameter, $R_a$ the axial resistivity, and $C_m$ the specific membrane capacitance. This yields a total of 60 compartments.

Each compartment contains Hodgkin–Huxley (HH) conductances: sodium, potassium, and leak. The sodium current, however, uses a three-gate formulation with gating vector $\lambda_{\text{Na}} = (m,h,s) \in [0,1]^3$. The

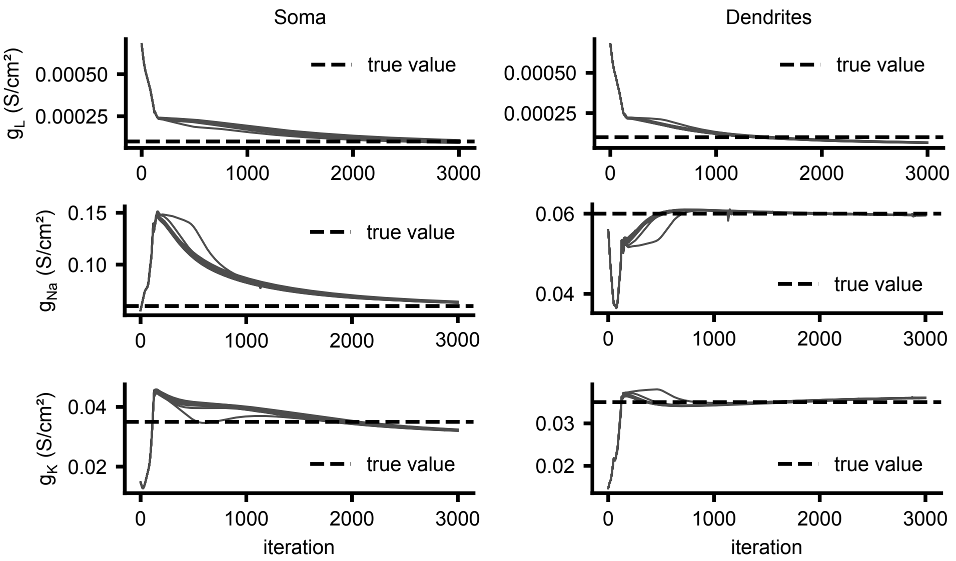

*Figure D.1:* **Inference of retinal ganglion cell (RGC) properties given multi-electrode array (MEA) recordings.** Estimation of all the maximum conductances for 10 optimization runs under noise level $\sigma_{\text{obs}} = 0.1\mu\text{V}$. All the maximum conductances were recovered accurately, with minor bias occasionally.

| **Location** | $m$ | $h$ | $n$ |
|---|---|---|---|
| Soma/Axon | $m_\infty = \frac{1}{1+e^{\frac{V+44}{-3}}}$ | $h_\infty = \frac{1}{1+e^{\frac{V+49}{3.5}}}$ | $n_\infty = \frac{1}{1+e^{\frac{V+46.3}{-3}}}$ |
| | $\tau_m = 0.05$ ms | $\tau_h = 1.0$ ms | $\tau_n = 3.5$ ms |
| Dendrite | $m_\infty = \frac{1}{1+e^{\frac{V+40}{-3}}}$ | $h_\infty = \frac{1}{1+e^{\frac{V+45}{3}}}$ | $n_\infty = \frac{1}{1+e^{\frac{V+42}{-2}}}$ |
| | $\tau_m = 0.05$ ms | $\tau_h = 0.5$ ms | $\tau_n = 2.2$ ms |

*Table 12:* Steady-state functions and time constants for gates $m$, $h$, and $n$ in the C10 model.

total transmembrane current released by a compartment is given by:

$$I_m = g_{\text{Na}}\, m^2 hs(V - E_{\text{Na}}) + g_{\text{K}}\, n^2(V - E_{\text{K}}) + g_{\text{L}}(V - E_{\text{L}}). \tag{9}$$

Moreover, unlike the canonical HH model, the gating kinetics vary across anatomical regions [Tomko et al., 2021]. Gating variables evolve according to first-order kinetics:

$$\tau_\lambda(V)\frac{d\lambda}{dt} = \lambda_\infty(V) - \lambda.$$

The steady-state functions $\lambda_\infty(V)$ and time constants $\tau_\lambda(V)$ for $m$, $h$, and $n$ vary by region and are listed in Table 12.

The third gating variable – $s$ – models sodium attenuation. Its dynamics are consistent across regions

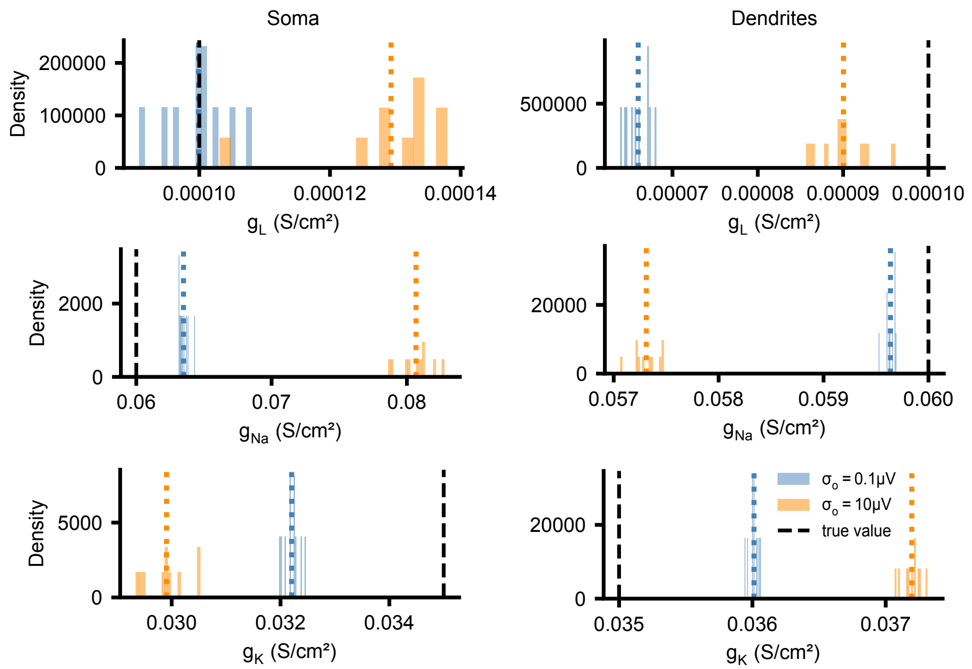

*Figure D.2:* **Impact of the observation noise on inference for the RGC model.** Distributions of inferred maximum conductances for $\sigma_{\text{obs}} \in \{0.1, 10\}\,\mu$V (colored dashed lines indicate the means). Increasing the observation noise generally led to higher bias and variance of the estimates, though the inferred values remained close to the ground truth.

| s | Formula |
|---|---|
| $s_\infty(V)$ | $\dfrac{1 + \text{Na\_att} \cdot e^{(V+60)/2}}{1 + e^{(V+60)/2}}$ |
| $\tau_s(V)$ | $\max\left(\dfrac{e^{0.0854(V+60)}}{0.0003(1 + e^{0.427(V+60)})}, 3.0\right)$ |

*Table 13:* Kinetics of sodium attenuation gate $s$ as a function of attenuation factor Na_att in the C10 model.

except for a region-specific attenuation factor Na_att $\in [0, 1]$. The expressions for $s_\infty(V)$ and $\tau_s(V)$ at fixed Na_att are given at temperature $T = 34°C$ (Table 13).

The attenuation factor `Na_att` is defined per section based on Migliore et al. [1999] (Table 14).

Reversal potentials follow Cutsuridis et al. [2010], with a small adjustment to $E_\text{L}$ to enforce a resting potential near −70 mV across compartments (Table 15).

The maximum conductances for each region were taken directly from Cutsuridis et al. [2010] (Table 16).

We used the following physiological constants: axial resistivity $R_a = 150\ \Omega \cdot \text{cm}$, specific membrane capacitance $C_m = 1\ \mu\text{F/cm}^2$, and extracellular resistivity $\rho_{\text{ext}} = 300\ \Omega \cdot \text{cm}$. While we assume homoge-

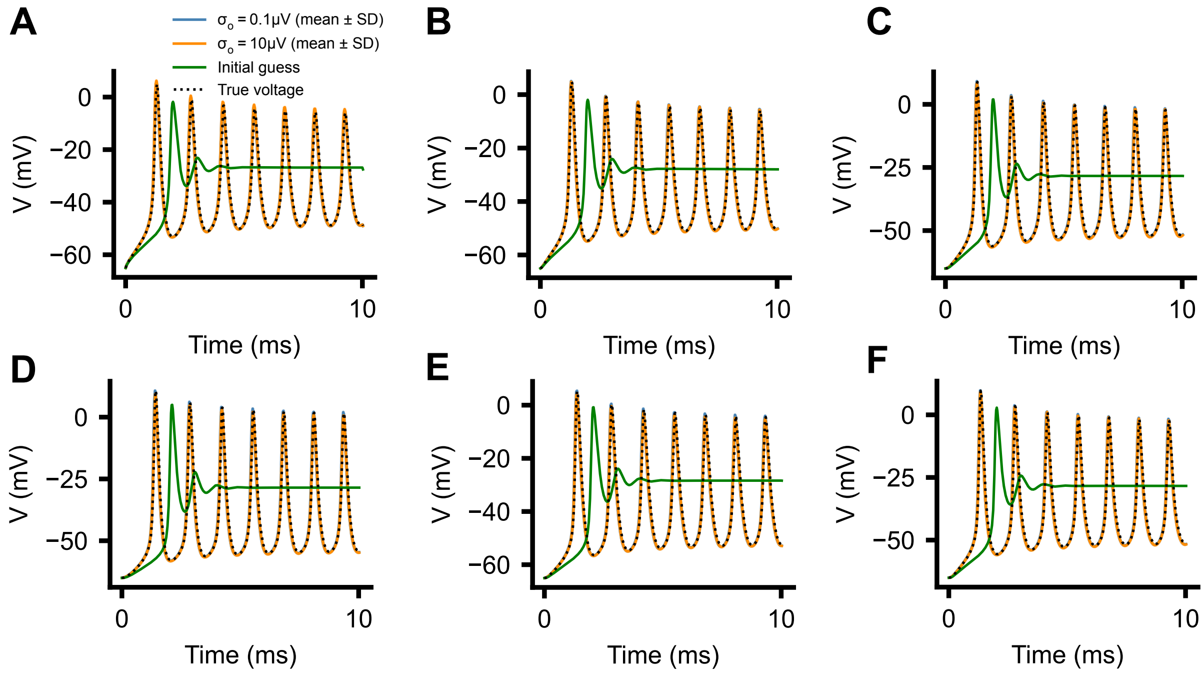

*Figure D.3:* **Membrane voltage recovery for the RGC model in six compartments.** **A**: soma, **B**: compartment 31, **C**: compartment 61, **D**: compartment 91, **E**: compartment 106, **F**: compartment 122. The ordering of the compartments is determined by the SWC file. For both low and higher noise levels, the membrane voltage was recovered accurately across the cell.

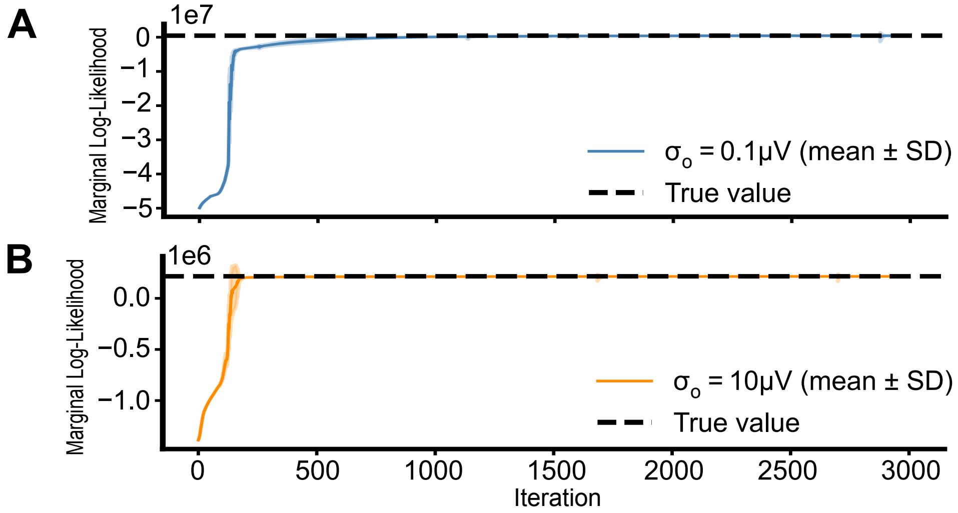

*Figure D.4:* **Marginal log-likelihood trace during optimization across 10 runs for the RGC model.** **A**: $\sigma_{obs} = 0.1\mu\text{V}$, **B**: $\sigma_{obs} = 10\mu\text{V}$. The marginal log-likelihood converged to its optimal value for both noise regimes.

neous extracellular resistivity, this simplification neglects known spatial variability [Gold et al., 2006], which could be addressed in future work. Note that Cutsuridis et al. [2010] also consider additional channels, which we neglected here. Including them in our model could also be the subject of future

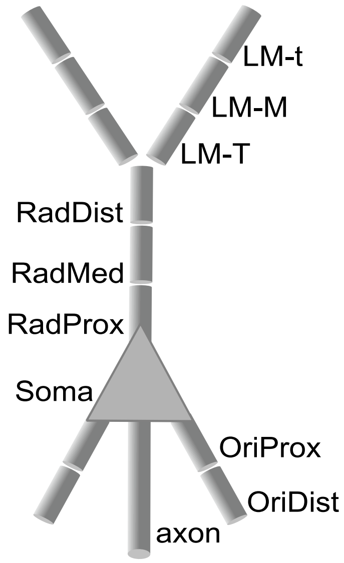

*Figure E.1:* **Morphology of the C10 model.** LM-t: lacunosum moleculare (thin), LM-M: lacunosum moleculare (medium), LM-T: lacunosum moleculare (thick), RadDist: radiatum distal, RadMed: radiatum medial, RadProx: radiatum proximal, OriProx: oriens proximal, OriDist: oriens distal.

| Section | Diameter (μm) | Length (μm) | Na_att |
|---|---|---|---|
| Soma | 10 | 10 | 0.8 |
| Axon | 1 | 150 | 1.0 |
| RadProx | 4 | 100 | 0.5 |
| RadMed | 3 | 100 | 0.5 |
| RadDist | 2 | 200 | 0.5 |
| OriProx | 2 | 100 | 1.0 |
| OriDist | 1.5 | 200 | 1.0 |
| LM-T | 2 | 100 | 0.5 |
| LM-M | 1.5 | 100 | 0.5 |
| LM-t | 1 | 50 | 0.5 |

*Table 14:* Detailed morphology of the C10 model and sodium channel attenuation (`Na_att`) per section.

| | Na | K | Leak |
|---|---|---|---|
| Reversal potential (mV) | 50.0 | −80.0 | −123.0 |

*Table 15:* Reversal potential in the C10 model.

experiments.

Synthetic extracellular recordings were generated by injecting a constant current of 1.0 nA into the soma for 30 ms. Voltage traces were simulated using a synthetic Neuropixel Ultra probe at a 40 kHz sampling rate, with noise standard deviation $\sigma_{obs} = 1\mu$V.

| **Region** | $g_L$ | $g_{Na}$ | $g_K$ |
|---|---|---|---|
| Soma | 0.0002 | 0.007 | 0.0014 |
| Axon | 0.000005 | 0.1 | 0.02 |
| OriProx | 0.000005 | 0.007 | 0.000868 |
| OriDist | 0.000005 | 0.007 | 0.000868 |
| RadProx | 0.000005 | 0.007 | 0.000868 |
| RadMed | 0.000005 | 0.007 | 0.000868 |
| RadDist | 0.000005 | 0.007 | 0.000868 |
| LM | 0.000005 | 0.007 | 0.000868 |

*Table 16:* Maximum conductances in the C10 model (in S/cm$^2$).

| **Region** | $g_{\mathrm{L}}$ | $g_{\mathrm{Na}}$ | $g_{\mathrm{K}}$ |
|---|---|---|---|
| Soma | [1e-4, 4e-4] | [0.003, 0.015] | [0.001, 0.005] |
| Axon | [1e-6, 1e-5] | [0.08, 0.12] | [0.015, 0.03] |
| Dendrite | [1e-6, 1e-5] | [0.003, 0.015] | [0.0005, 0.002] |

*Table 17:* Initialization bounds for the maximum conductances in the C10 model (in S/cm$^2$).

The true location of the cell was generated by applying a rigid-body transformation—consisting of a 3D rotation and translation—to the positions of the compartment centers defined via the SWC file. The geometric parameters thus included three rotation angles (one for each axis), applied to the base positions of the compartment centers obtained from the SWC file, and a 3D translation vector applied to the base position of the soma compartment.

We fit this model using standard full covariance EKF. All voltages and channel states were initialized at their steady-state values at rest, and thus we assumed the initial state to be known. We set the initial covariance matrix to $\Sigma_0 = 0.1I$. Observation noise was also assumed to be known, as commercial devices that record extracellular potentials typically provide noise specifications [Ye et al., 2024, Multi Channel Systems MCS GmbH, 2020]. Since the state dynamics were well-specified and deterministic, we fixed the standard deviation of the membrane potentials and channel states to small values: $\sigma_v = 0.001\,\mathrm{mV/ms}^{1/2}$, and $\sigma_{\mathrm{state}} = 0.0001$ ms$^{-1/2}$ (same for all the channels) for all compartments. These values ensured numerical stability while avoiding degenerate gradients during optimization.

We optimized maximum conductances and geometric parameters using the Adam optimizer. We tried multiple learning rates and initialized the maximum conductances uniformly at random within the bounds shown in Table 17. The rotation angles were initialized uniformly on $[0, 2\pi)$, and translations were initialized to place the soma near its true location. We chose this initialization to reduce the risk of converging to a mirror-reflected solution, which produces identical extracellular signals when using a planar probe (see Section C). We ran 100 random initializations with different seeds and selected the result that achieved the highest marginal log-likelihood.

Although the marginal log-likelihood converged to its true value after a few hundred iterations (fig. E.3F), and both the voltage traces (fig. E.3A–E) and compartment positions (fig. 5A) were accurately recovered across the cell, some maximum conductances did not converge to their true values or even continued to

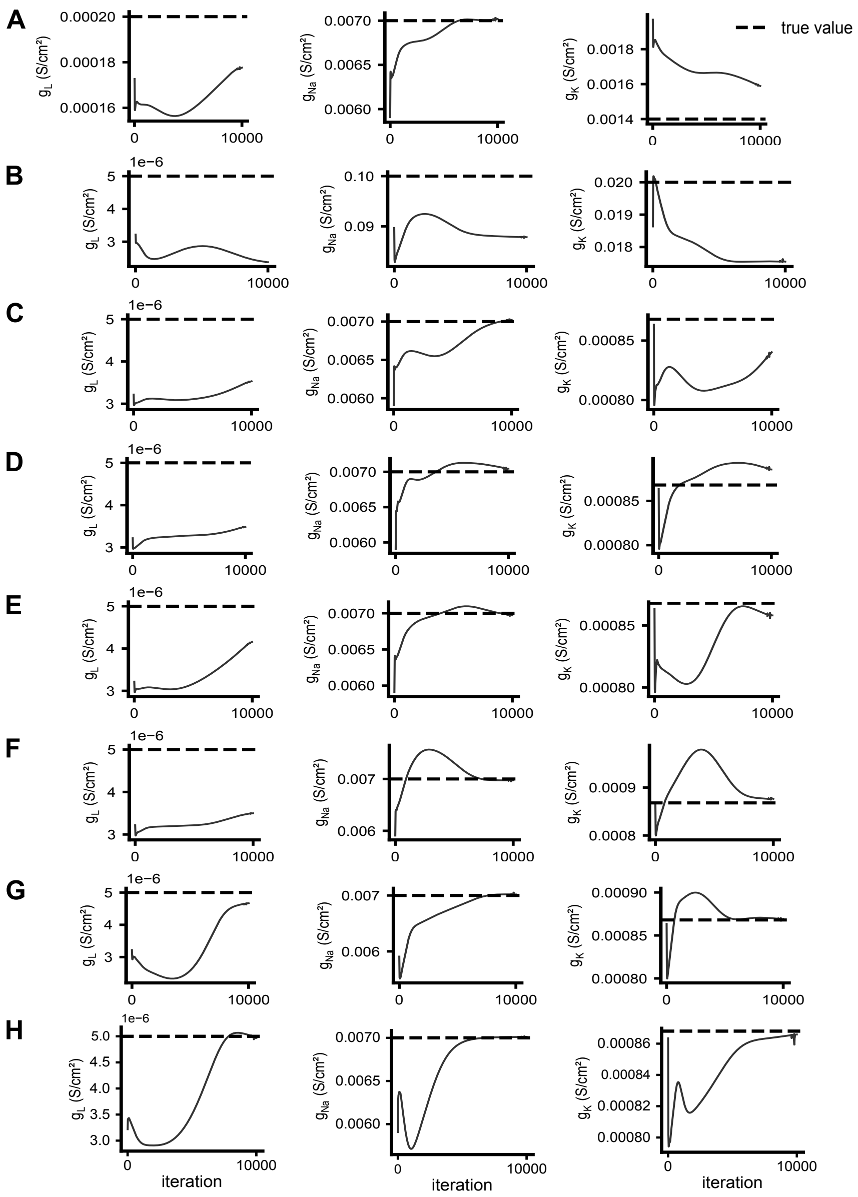

*Figure E.2:* **Inference of the maximum conductances of the C10 model in all the regions.** **A**: Soma, **B**: Axon, **C**: OriProx, **D**: OriDist, **E**: RadProx, **F**: RadMed, **G**: RadDist, **H**: LM. While several maximum conductances converged to their true values, many were still fluctuating.

fluctuate (fig. E.2) —reflecting the well-known degeneracy in conductance-based neuron models, where multiple parameter combinations can produce nearly identical voltage dynamics.

## F DiagEKF vs. EKF

We compare the computational efficiency of the standard Extended Kalman Filter (EKF) and our sparse diagonal approximation (DiagEKF). To isolate performance differences, we consider a simplified neuron

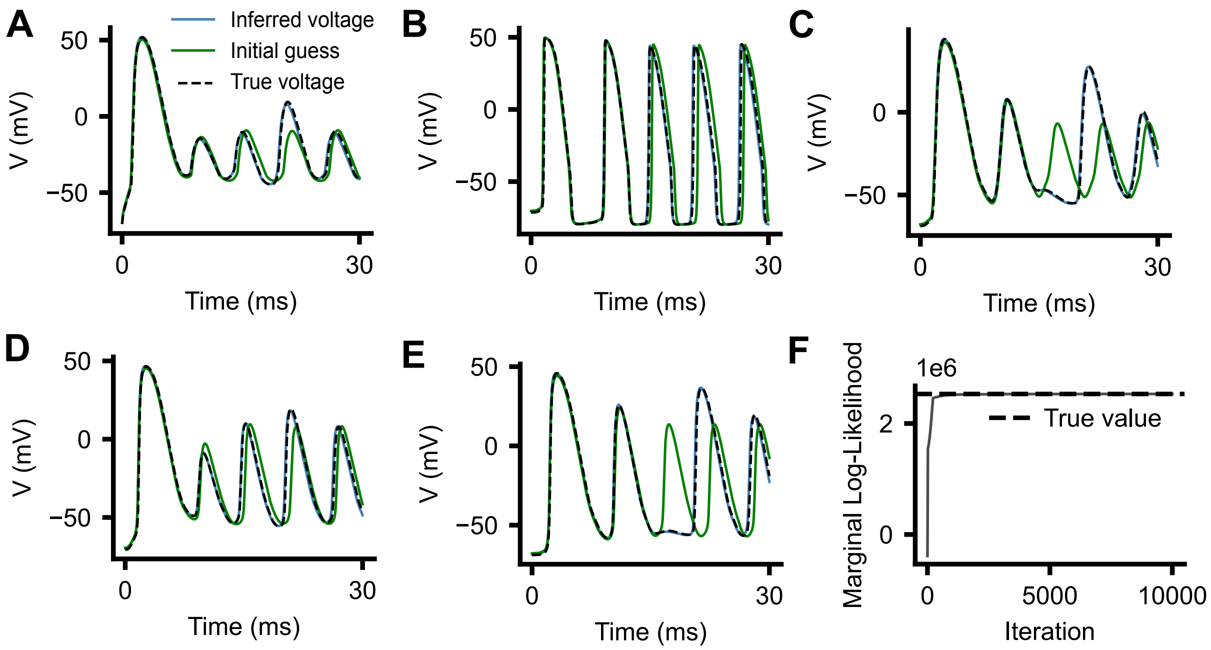

*Figure E.3:* **Recovery of the membrane voltage in different regions of the pyramidal cell.** **A**: Soma, **B**: Axon, **C**: RadDist, **D**: OriDist, **E**: LM. **F**: Marginal log-likelihood trace during optimization. Despite some maximum conductances failing to converge to their true values, the membrane voltage was recovered accurately across the cell and the marginal log-likelihood converged to its true value, reflecting the well-known degeneracy in conductance-based neuron models.

model: a 24 $\mu$m cable equipped with standard Hodgkin–Huxley channels [Hodgkin and Huxley, 1952]. A constant current is injected for 20 ms, and extracellular voltage is recorded at a single electrode at a 40 kHz sampling rate.

The neuron is discretized into $N$ compartments, with $N \in \{1, 10, 20, 50, 100, 200\}$. As $N$ increases, the number of state variables (membrane voltages and gating variables) grows proportionally. Importantly, the gating dynamics in these models are highly local: each variable typically depends only on its own previous value and the voltage of its corresponding compartment. As a result, the Jacobian of the dynamics function quickly becomes very sparse when the number of states increases (fig. F.1A).

To evaluate performance, we ran both EKF and DiagEKF for 500 iterations on an NVIDIA H100 GPU for each value of $N$. As shown in fig. F.1B, DiagEKF achieves approximately 2× speedup over the dense EKF in high-dimensional settings. This highlights the computational advantage of our diagonal approximation in realistic biophysical neuron models.

In our work, we used DiagEKF to fit extracellular voltage recordings simulated from a retinal ganglion cell. All the other experiments were performed using standard EKF.

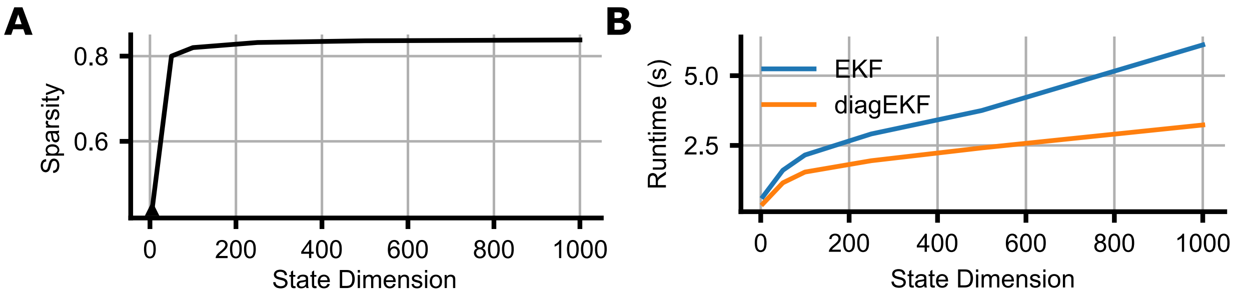

*Figure F.1:* **Computational efficiency of our diagonal approximation to the EKF filtering distribution A**: Sparsity of the Jacobian of the dynamics function over state dimension. Especially large biophysical models are sparse, suggesting that sparse approximations to the filtering covariance could provide accurate but efficient inference. **B**: Average runtime over 500 updates for the EKF and sparse diagEKF versus state dimension for a 20*ms* time horizon. The EKF with diagonal approximation is twice as fast as EKF with a dense covariance for large models.
